# Association Between Mobile App Use and Caregivers’ Support System, Time Spent on Caregiving, and Perceived Well-being: Survey Study From a Large Employer

**DOI:** 10.2196/28504

**Published:** 2022-04-11

**Authors:** Pelin Ozluk, Rebecca Cobb, Alyson Hoots, Malgorzata Sylwestrzak

**Affiliations:** 1 HealthCore Inc Wilmington, DE United States; 2 Anthem Inc Indianapolis, IN United States

**Keywords:** caregiving, mobile app, mobile phone

## Abstract

**Background:**

Mobile technology to address caregiver needs has been on the rise. There is limited evidence of effectiveness of such technologies on caregiver experiences.

**Objective:**

This study evaluates the effectiveness of ianacare, a mobile app, among employees of a large employer. ianacare mobilizes personal social circles to help with everyday tasks. Through the use of ianacare, we evaluate the associations between coordinating caregiving tasks among a caregiver’s personal support network and outcomes related to the caregiver’s support system, time use, perceived productivity, and perceived health and well-being. Caregiver tasks include tasks such as meal preparation, respite care, pet care, and transportation. Time use is the measure of a caregiver’s time spent on caregiving tasks and how much time they had to take off from work to attend planned or unplanned caregiving tasks.

**Methods:**

We conducted 2 surveys to assess within-participant changes in outcomes for the unpaid, employed, caregivers after 6 weeks of using the mobile app (n=176) between March 30, 2020, and May 11, 2020. The surveys contained questions in three domains: the caregiver’s support system, time use and perceived productivity, and perceived health and well-being. The results of the linear probability models are presented below.

**Results:**

App use was significantly associated with decreasing the probability of doing most caregiving tasks alone by 9.1% points (SE 0.04; *P*=.01) and increasing the probability of at least one person helping the primary caregiver by 8.0% points (SE 0.035; *P*=.02). App use was also associated with improving the time use of the primary caregiver who took significantly less time off work to attend to caregiving duties by 12.5% points (SE 0.04; *P*=.003) and decreased the probability of spending more than 30 hours weekly on caregiving by 9.1% points (SE 0.04; *P*=.02). Additional findings on the positive impact of the app included a decrease in the probability of reporting feeling overwhelmed by caregiving tasks by 12.5% points (SE 0.04; *P*=.003) and a decrease in the probability of reporting negative health effects by 6.8% points (SE 0.04; *P*=.07) because of caregiving. Although subjects reported that COVID-19 increased their stress attributed to caregiving and prevented them from requesting help for some caregiving tasks, using the app was still associated with improvements in receiving help and lessening of the negative effects of caregiving on the caregivers.

**Conclusions:**

App use was associated with improvements in 7 of 11 caregiver outcomes across three main categories: their support system, time spent on caregiving, and perceived health and well-being. These findings provide encouraging evidence that the mobile app can significantly reduce caregiver burden by leveraging a caregiver’s support network despite the additional challenges brought by COVID-19 on caregivers.

## Introduction

### Background

In the United States, caregiver burden is a rising problem with a rapidly growing senior population. The number of Americans providing unpaid care has increased from 43.5 million to 53 million over the past 5 years [1]. Although people of all demographics (eg, young children and spouses with health problems) may need caregiving, older adults constitute a large proportion of care recipients. A growing population of older adults with longer life expectancies means that the number of people needing caregiving in the future is likely to continue to increase [1]. Caregiving encompasses a broad range of activities that often depend on the severity of the care recipient’s diagnosis and condition [2]. In addition, the relationship between family caregivers and care recipients can determine the types of activities required. For example, taking care of a parent with mobility issues will be different from taking care of a spouse with cancer. Caregiving is associated with several negative health outcomes such as mental distress, poor self-care, sleep deprivation, and caregiver burden, which are often overlooked by clinicians [1,3]. In the remaining part of the paper, a caregiver is defined as any unpaid family caregiver who may be taking care of a relative or a friend. Caregiver burden may also negatively affect work-life balance. Approximately 10% of caregivers had to give up work entirely or retire early [1].

The literature finds that whether the burden associated with caregiving is subjective (ie, self-reported outcomes of happiness, health status, and quality of life) or objective (ie, outcomes of time spent, expenses, and taking care of daily tasks) depends on the care recipient’s condition and caregiver characteristics [4-6]. For example, caring for a person living with severe physical disabilities or schizophrenia was found to be associated with subjective burden on the caregiver, although the link between these conditions and the objective burden was not clear [5,6]. Furthermore, decreased social activity and feelings of isolation as one takes the role of caregiver can exacerbate the burden of caregiving, leading to poorer physical and mental health [3]. Research also shows caregiver characteristics, such as being female or living with the care recipient, may also contribute to caregiver burden [7,8]. Another study found that perceived social support may be more consistently related to subjective burden than the actual received social support [9].

Asking for help may be especially difficult for primary caregivers. Often, they end up taking on the entire burden of caregiving, even though other resources such as community services or other family members or friends are available and willing to help [1,3]. There are many reasons why caregivers may not ask for help: financial concerns, fear of losing their privacy, or feelings of shame regarding care needs [3]. One hypothesized link between social connections and health is that the social support people receive from their network of friends and loved ones may *buffer* against the detrimental physical consequences of psychosocial stress. Increased social support is found to be associated with a decrease in caregiver burden [10].

Although most of the literature focuses on caregiving burden, research has also pointed out that there are positive aspects attributed to caregiving [7,11-13]. Specifically, caregiving can bring opportunities such as being able to give back, discovering personal strength, becoming closer to the care recipient, and gaining a sense of accomplishment and competence.

Owing to the growing population of caregivers, there is an increasing interest in technological innovations to ease caregiving burden. Web-based interventions among caregivers appear to be focused on certain diagnosis groups (ie, dementia and chronic conditions) [14-20]. Most of the web-based interventions evaluated provide information, education, and peer or professional support. A review of web-based interventions to improve caregiver health and general caregiving outcomes found significant reductions in stress or distress because of technological interventions. However, results from the evaluations of such interventions were mixed owing to small sample sizes or weak study designs [15].

Although many studies have examined the efficacy of web-based interventions, our knowledge on the role of mobile apps designed for caregiver burden is relatively limited [21-23]. Ghahramani and Wang [21] investigated predictors of caregivers’ willingness to adopt caregiving-related mobile apps. They found that caregivers’ capabilities and skills in using an app, an app’s effectiveness in responding to the caregivers’ needs, and the degree of control caregivers had over their responsibilities were factors that affected the willingness of caregivers to adopt caregiving-related mobile apps. As a care recipient’s health was perceived to be more severe, caregivers reported being more likely to use an app. In addition, as the threat of unexpected health changes became more likely, caregivers reported perceiving an app as a more efficient tool [21].

Our study aims to evaluate the effectiveness of a mobile app in decreasing caregiver burden and increasing perceived support and well-being among employees of a large employer. The authors of the paper were employed by the large employer during the study period and dissemination of study results. The mobile app works by connecting the unpaid, primary caregiver to his or her personal social support group (ie, friends, family members, and community services) in a convenient way, which helps to lower the barrier in requesting and offering support. In addition, the app provides the caregiver with access to multiple resources and tools. Specifically, this study contributes to the literature in three ways. First, we evaluate the impact of a mobile app specifically designed for unpaid caregivers—a topic that has not been widely studied but hypothesized to reduce burden [21-23]. The app’s specific function is to make it easier to coordinate help for the caregiver. Through this focus, we can better understand how the app positively affects the caregiver and decreases the burden. Second, we evaluate outcomes that were not typically reported in previous studies, such as the impact of caregiving on perceived work productivity and time use. Finally, we provide suggestive evidence on how a public health crisis, such as COVID-19, affects caregiver burden and how the app is able to decrease caregiver burden despite unforeseen factors and limitations.

### Caregiver App

The ianacare app was selected through a competition among technology start-ups as a part of a large employer’s strategy to use technology that aims to improve health and well-being across its employees. Ianacare was 1 of 5 companies selected from a pool of 126 applicants. Applications from start-ups were judged based on the technical implementation feasibility and merit of the proposed solution.

The app was launched in 2019. Being family caregivers themselves, the founders observed that help was often not exchanged between the caregiver and their support groups owing to the feeling of intense burden on behalf of the caregiver. The ianacare mobile app was designed to leverage technology and act as an effective buffer to organize and mobilize social networks around caregivers; the *iana* of ianacare stands for *I Am Not Alone*.

The mobile app allows a support team to be mobilized when the caregiver makes needs known, such as coordinating physician visits, dropping off groceries, and medication delivery. This mobilization allows the burden of care to be distributed among more people and makes it easier to coordinate schedules, while providing a platform for sharing emotional support. Once the app is downloaded, the caregiver is asked to create his or her team of supporters by inviting them to download the app and join the caregiver’s support team. When there is help needed for a specific caregiving task, the caregiver can click on the appropriate icon of available options (ie, errands, check-in visits, meals, rest or breaks, pet care, childcare, rides, and other events). After choosing one of the help options, the caregiver can specify the person or persons that he or she wants help from. They also specify the locations, dates, and time on a calendar visible by the caregiver’s support group, as well as any other specifications. Once help is requested, a notification is sent, and the caregiver is notified when someone accepts the request. Both caregivers and care recipients can request and accept help, post updates, and invite or remove supporters. Caregivers and support group members can also post updates and pictures keeping each other engaged in the care recipient’s situation confidentially on the app. One of the advantages of ianacare is the built-in choice for requesting different types of help that can be sent to a caregiver’s team of supporters all at once, eliminating the need to manage one-on-one conversations. Team members can see who helped on which tasks, which can lead to a more even distribution of caregiving tasks among the team. Our hypothesis is that caregivers using the app will be more likely to ask and receive help from their support system, reducing the burden associated with having to tailor each help request as individual conversations whenever help is needed. When requesting help becomes easier, caregivers will be more likely to seek it. This leads to improvements in other domains such as work productivity, well-being, leisure time, and a general feeling of support.

This study attempts to fill the gap in the literature on this topic by evaluating the impact of the app on a sample of unpaid, employed caregivers using a pre- and postsurvey design that asks questions on both objective and subjective outcomes of caregiving. We focus on three primary outcomes for the primary caregiver: (1) support system, as defined by the availability of helpers to the caregiver, ease of assistance with caregiving tasks, and feelings of being supported as a caregiver; (2) time use and productivity outcomes, defined as hours spent on caregiving tasks, caregiving tasks that affect work hours, perceived productivity at work, and perceived impact of caregiving on caregiver’s work; and (3) well-being, defined as the frequency of feeling overwhelmed by caregiving tasks and the impact of caregiving on the caregiver’s perceived health [4,24].

## Methods

### Survey Implementation

We used a pre- and postsurvey design to measure the effectiveness of the app. The app was offered from March 30, 2020, to April 13, 2020, to employees of a large national employer. The employer selected ianacare based on their interest in helping alleviate caregiver burden among their employees. The survey was developed by a study team including the authors of this paper as well as internal company survey data collection experts.

Employees were encouraged to download and use the app for a 6-week period on the announcement of the employer’s intranet. Inclusion criteria included: being an unpaid primary caregiver for at least one person, having a smartphone or tablet, and being with the same employer during the 6 weeks between the first and second surveys. Eligible participants completed a web-based 10-minute survey at the time of enrollment. If they were the primary caregiver for more than one person, we asked participants to answer questions only for the person for whom they spent the most time providing care. After 6 weeks of app use, participants were invited by email to take the second web-based survey, which took approximately 10 minutes to complete. A gift card of US $25 was provided upon the successful completion of both surveys. Results from both surveys remained anonymous and were aggregated without individual names. Survey recruitment materials and the informed consent section emphasized the voluntary nature of participation and the option to end participation at any time without penalty or loss of benefits. Participants wishing to withdraw from the study were able to communicate their requests via email. After withdrawal, the participant would no longer receive any study-related communication and the study team would remove their data. We also excluded survey participants if they were never active on the app.

The pre- and postapp use surveys consisted of questions about participants’ demographics (age and gender), socioeconomic status (education and income levels), and caregiving characteristics (person being cared for, where person resides, and types of caregiving tasks). The preapp use survey also provided a baseline assessment of the caregiver’s support system and the impact of caregiving on perceived productivity and health. The survey question which the health outcome was generated from does not distinguish between physical and mental health. The postapp use survey included identical questions from the first survey. The postapp use survey also included questions on the mobile app’s impact on the caregiving burden of participants, their opinions about existing app features, and other potential features that could be added to the app in the future. Given that the study timeline overlapped with the emergence of COVID-19 in 2020, the postapp use survey also included questions on the impact of COVID-19 on their caregiving situation.

The surveys included both objective and subjective questions. We labeled questions where the respondent’s answer could be determined as right or wrong as objective and those questions where the respondent’s answer pertained to their perceptions and feelings as subjective.

### Ethical Considerations

An institutional review board review was not needed because data were collected as part of the employer’s quality improvement initiative (as opposed to human subject research) in an effort to improve benefits offered to employees. For the full survey questionnaire, please refer to Multimedia Appendix 1.

### Survey Questions

#### Caregiver’s Support System

Information pertaining to the caregiver’s support system data was based on questions in both the pre- and postapp use surveys about the proportion of caregiving tasks performed by the participant and about the frequency of feeling supported as a caregiver.

#### Time Spent on Caregiving and Perceived Productivity at Work

All participants were asked identical questions before and after app use on the time spent on caregiving and their perception of how caregiving impacted their productivity at work. Participants were asked to report hours spent per week in the past month on caregiving (eg, food prep, care assistance, coordinating physician visits, and grocery shopping) and hours they needed to take off from work owing to planned caregiving tasks (eg, physician appointments) and unplanned caregiving tasks (eg, medical emergencies). Participants were asked to assess how they felt their caregiving role impacted their productivity or focus at work. In addition, they were asked if they felt caregiving put them at a disadvantage compared with coworkers in terms of work performance and recognition.

#### Caregiver Well-being

All participants were asked identical questions regarding their overall well-being as caregivers over the past 30 days, including their stress level and perceived caregiver burden, both before and after using the mobile app. Specifically, participants were asked to report the frequency of feeling overwhelmed by caregiving tasks (eg, worrying about the person they were caring for) and how often they felt caregiving negatively impacted their own health in the past 30 days (eg, caregiver missing their own physician appointments or missing their own medicine because they were too busy caring for someone else).

#### Impact of COVID-19 on Caregiving

As the study period overlapped with the 2020 COVID-19 pandemic, participants were asked to assess the impact of COVID-19 on their caregiving role. A 5-point Likert scale was used to scale responses. Participants were asked questions regarding their productivity in terms of caregiving hours related to COVID-19, including how COVID-19 affected the number of hours spent per week on caregiving, whether COVID-19 increased the amount of time needed to take off work to attend to caregiving tasks, participants’ productivity based on whether COVID-19 put them at a greater disadvantage at work owing to being a caregiver, and whether COVID-19 negatively impacted focus at work because of increased worry about caregiving tasks. The overall well-being of the participants was assessed using the question of how COVID-19 impacted stress levels around caregiving tasks.

### Outcome Measurement

We evaluated the app’s effectiveness in three outcome areas: support system, time use and productivity, and well-being. Outcomes related to a caregiver’s support system were assessed with binary indicators: (1) whether the respondent reported doing more than half of the caregiving by himself or herself during the past 30 days, (2) whether the respondent reported having no one supporting him or her in caregiving tasks during the past 30 days, and (3) whether the respondent reported *never* or *almost never* to feeling supported by his or her social network during the past 30 days. Outcomes of caregiving time use and productivity were assessed using binary indicators, including: (1) whether the respondent reported spending more than 30 hours weekly on caregiving tasks in the past 30 days, (2) whether the respondent reported that he or she needed to take any time off from work owing to planned caregiving tasks in the past 30 days, (3) whether the respondent reported he or she unexpectedly needed to take any time off from work owing to caregiving tasks, (4) whether the respondent reported his or her work productivity and focus to be negatively affected by caregiving, and (5) whether the respondent reported caregiving put him or her at a disadvantage compared with coworkers in terms of work performance and recognition. Outcomes of well-being were assessed using binary indicators for the probability of reporting (1) fairly or very often feeling overwhelmed by caregiving tasks in the past 30 days and (2) fairly or very often feeling negative health effects related to caregiving.

### Statistical Analyses

To measure the association between app use and caregiving outcomes, we compared pre- and postsurvey responses and reported within-participant changes in outcomes using linear probability models. In addition, we compared the distribution of respondents’ full scale of answers without turning them into binary variables. For the distributional analyses, we present boxplots to show the distribution of the answers to the questions assessing outcomes of support system, time use and productivity, and self-reported well-being. We also reported the average within-participant change in the full scale of answers before and after app use. Power analysis was not conducted.

## Results

### Overview

**Figure 1:**
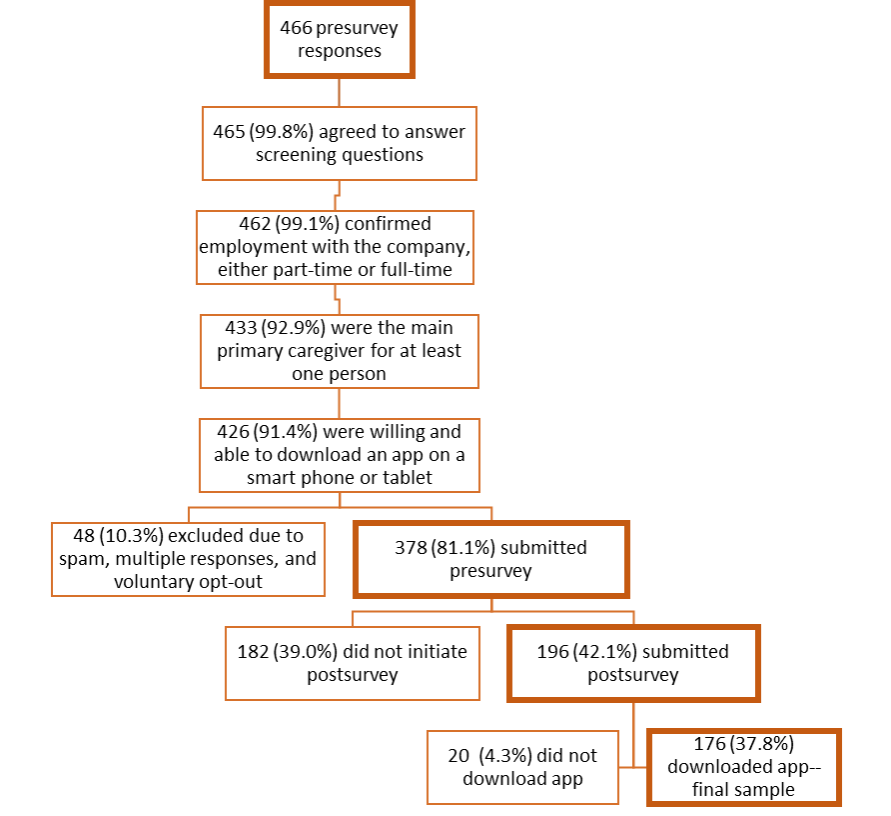
Sample construction.

The final sample included 176 individuals who responded to the pre- and postapp surveys (Figure 1). Our sample was predominantly female (167/176, 94.9%), with an average age of 47 (SD 9.71) years. More than half of the participants were White, college educated, and had at least US $75,000 in household income. Most participants reported taking care of their parents, living with the person they were caring for, and providing daily living and administrative assistance (ie, food prep, housekeeping, managing insurance, coordinating physician visits, and financial management; Table 1).

**Table 1:** Characteristics of individuals in the final sample (N=176).

Predictors		Values, mean (SD)	Value, n (%)
Age (years)		46.99 (9.71)	176 (100)
Female		0.95 (0.22)	167 (94.9)
Married		0.50 (0.50)	88 (50)
**Race**			
	White	0.55 (0.50)	96 (54.5)
	Black	0.24 (0.43)	42 (23.9)
	Hispanic or Latino	0.10 (0.30)	18 (10.2)
	Asian	0.04 (0.20)	7 (4)
	American Indian or Alaska native	0.01 (0.08)	2 (1.1)
	Native Hawaiian or other Pacific islander	0.01 (0.08)	2 (1.1)
	Other	0.01 (0.11)	2 (1.1)
	Prefer not to respond	0.04 (0.20)	7 (4)
**Education**			
	High school	0.04 (0.20)	7 (4)
	Some college	0.20 (0.40)	35 (19.9)
	Associate degree	0.22 (0.42)	39 (22.2)
	Bachelor’s degree	0.23 (0.42)	40 (22.7)
	Graduate degree or higher	0.30 (0.46)	53 (30.1)
	Prefer not to respond	0.01 (0.08)	2 (1.1)
**Categories of annual household income (US $)**			
	<50,000	0.18 (0.38)	32 (18.2)
	50,000-75,000	0.07 (0.25)	12 (6.8)
	75,000-100,000	0.17 (0.38)	30 (17)
	100,000-150,000	0.24 (0.43)	42 (23.9)
	>150,000	0.13 (0.33)	23 (13.1)
	Prefer not to say	0.21 (0.40)	37 (21)
Number of people in household		3.29 (1.23)	176 (100)
**Care recipient**			
	Parent	0.48 (0.50)	85 (48.3)
	Child under 18	0.32 (0.47)	56 (31.8)
	Other^a^	0.20 (0.40)	35 (19.9)
**Care recipient lives**			
	With the care recipient	0.63 (0.49)	111 (63.1)
	Alone	0.17 (0.38)	30 (17)
	Other	0.20 (0.40)	35 (19.9)
**Type of tasks done by caregiver**			
	Daily living assistance	0.69 (0.46)	121 (68.8)
	Care assistance	0.37 (0.48)	65 (36.9)
	Administration	0.64 (0.48)	113 (64.2)
	Other^b^	0.09 (0.28)	16 (9.1)

^a^Includes spouses, children aged >18 years, grandchildren, grandparents, partners, friends, housemates, coworkers, and neighbors.

^b^Includes web-based school assistance, home schooling, nurturing, assisting with socialization and emotional support.

We observed significant differences in 2 of the support system outcomes. Specifically, after using the app, the probability of caregivers reporting *doing more than half of all the caregiving tasks by themselves* decreased by 9.1% points (SE 0.036; *P*=.01). The probability of caregivers reporting *no one helping them in caregiving tasks* also decreased by 7.9% points (SE 0.035; *P*=.02). There was no evidence that the app had any impact on reducing the caregiving load for caregivers who reported doing all caregiving by themselves. We also did not observe any meaningful difference in perceived support by one’s support group (Table 2).

**Table 2:** Association of app use with caregiving outcomes (N=176).

	Support system					Time use and perceived productivity						Perceived health and well-being	
	Does all caregiving (objective)	Does more than half (objective)	No one helps (objective)	No network support (subjective)	More than 30 hours spent weekly (objective)		No planned time taken (objective)	No unplanned time taken (objective)	Negative effect on productivity (subjective)	Feeling disadvantaged at work (subjective)	Often feeling negative health effects (subjective)		Often overwhelmed (subjective)
App effect percentage point change associated with app use (SE)^a^	−0.0227 (0.0351)	−0.0909 (0.0354)	−0.0795 (0.0347)	0.0227 (0.0369)	−0.0909 (0.0398)		0.125 (0.0408)	0.125 (0.0439)	−0.0284 (0.0374)	0.017 (0.0296)	−0.0682 (0.0375)		−0.125 (0.0416)
Observations	352	352	352	352	352		352	352	352	352	352		352
*R* ^2^	0.002	0.036	0.029	0.002	0.029		0.051	0.044	0.003	0.002	0.019		0.049
*P* value	.52	.01	.02	.54	.02		.003	.005	.45	.57	.07		.003

^a^Robust SE.

In terms of time use and perceived productivity outcomes, we observed several improvements. After 6 weeks of app use, both the probability of reporting *taking no time off from work due to planned caregiving tasks* and the probability of reporting *taking no time off of work due to attend unscheduled caregiving tasks* increased by 12.5% points (SE 0.04; *P*=.003 and *P*=.005, respectively). We also observed that after 6 weeks of app use, there was a significant decrease in the probability of reporting *spending more than 30 hours weekly on caregiving tasks* by 9.1% points (SE 0.04; *P*=.02; Table 2).

The probability of reporting *often feeling negative health effects due to caregivin*g decreased by 6.8% points, although the estimate was only significant at a 10% significance level (SE 0.04; *P*=.07). After 6 weeks of app use, the probability of reporting *often feeling overwhelmed by caregiving tasks* decreased by 12.5% points (SE 0.04; *P*=.003; Table 2)**.**

To summarize, we observed improvements in 7 out of 11 caregiver outcomes. App use was significantly associated with decreasing the amount of caregiving tasks that fell on the primary caregiver and increasing the likelihood of at least one person helping him or her. Use of the app was also associated with improvements in the time management of the primary caregiver. Significantly less time was taken off work to attend to caregiving, and the likelihood of spending <30 hours weekly on caregiving was significantly decreased. There is also suggestive evidence that the app may be associated with decreasing feelings of being overwhelmed and improving the perceived impact of caregiving on caregivers’ health.

**Figure 2:**
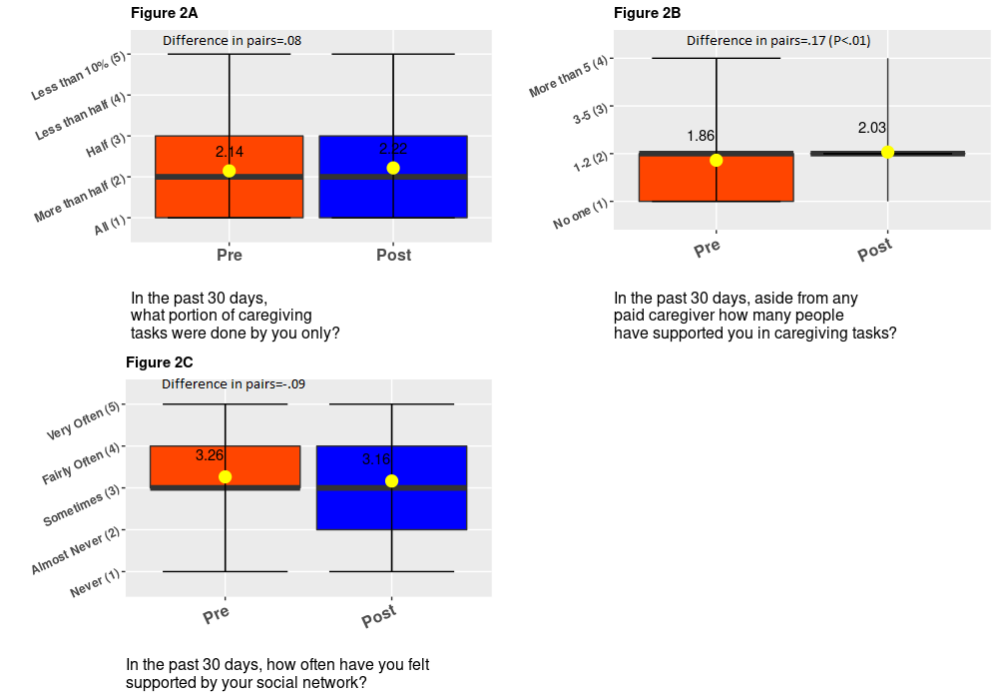
Distribution of responses to support system questions.

**Figure 3:**
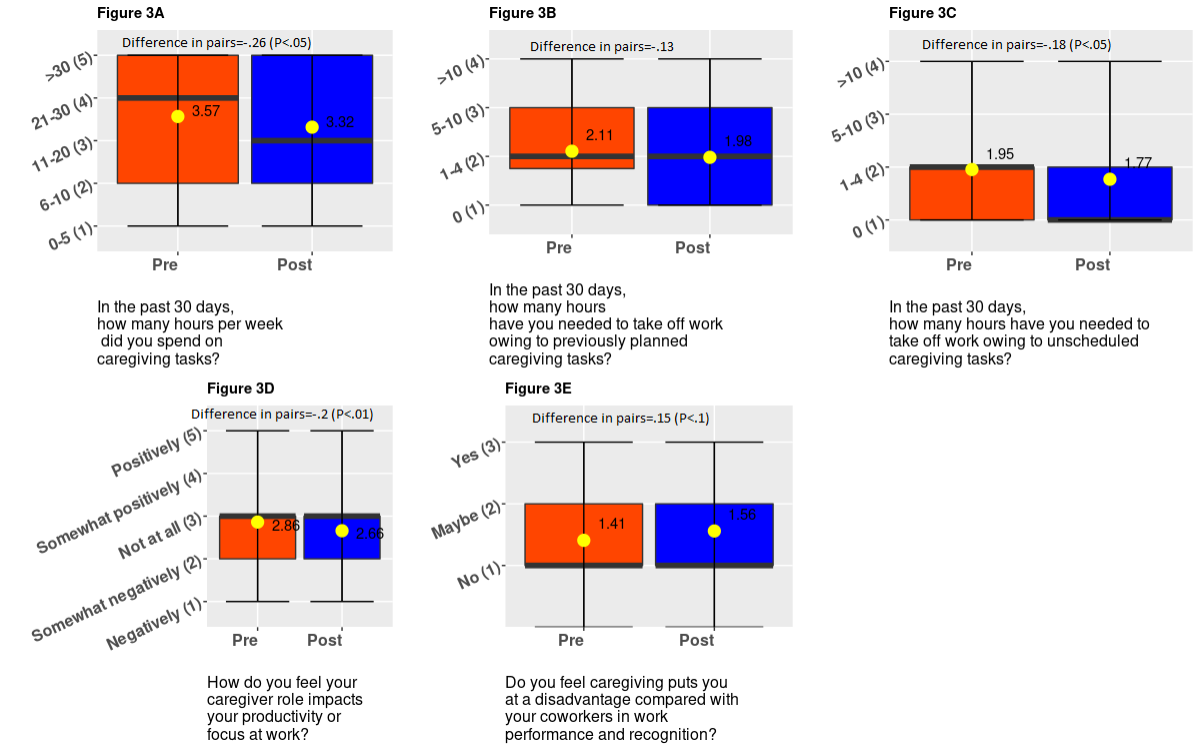
Distribution of responses to time use and perceived productivity questions.

**Figure 4:**
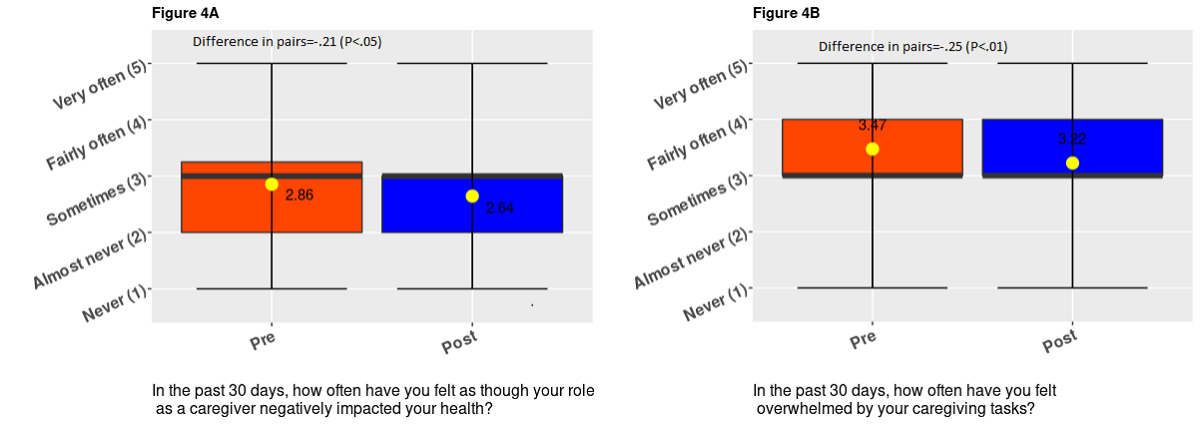
Distribution of responses to perceived health and well-being questions.

We observed that the responses to the number of people supporting the primary caregiver improved significantly (*P*=.006; Figure 2) with app use. We observed significant changes in responses regarding the number of hours spent on caregiving tasks per week (*P*=.04) and the number of hours taken off from work to attend to unscheduled caregiving duties (*P*=.03; Figure 3). These significant changes pertain to responses shifting toward decreasing hours of caregiving reported. We observed that responses to work outcomes significantly became worse; we observed declines in perceived work productivity or focus (*P*=.004), and feelings of being disadvantaged at work owing to caregiving increased (*P*=.06; Figure 3). However, there was a significant improvement in the perceived impact of caregiving on participants’ health (*P*=.02; Figure4). Finally, we observed a positive statistically significant reduction in participants’ feelings of being overwhelmed about caregiving after using the app (*P*=.004; Figure 4).

### Impact of COVID-19 Pandemic

A total of 35.2% (62/176) of the participants said that COVID-19 significantly increased their time spent on caregiving tasks (Table 3). Approximately 67% (118/176) said COVID-19 increased their stress attributed to caregiving, and 40.9% (72/176) reported that COVID-19 negatively impacted their focus at work. We also observed that most participants reported that COVID-19 prevented them from asking others to help, although we observed a significant increase both in the probability of acquiring help from at least one person and the number of people helping in caregiving. We interpret this as suggestive evidence that the app was useful in enabling caregivers to seek more help from others despite COVID-19 making it more difficult. Most participants reported that COVID-19 did not increase the need to take time off from work to attend to caregiving.

**Table 3:** Responses to questions about COVID-19’s impact (N=176).

Factors to assess COVID-19 impact	Significantly increased, n (%)	Somewhat increased, n (%)	No impact, n (%)	Somewhat decreased, n (%)	Significantly decreased, n (%)	Missing, n (%)
Caregiving time spent	62 (35.2)	35 (19.9)	34 (19.3)	10 (5.7)	15 (8.5)	20 (11.4)
Caregiving stress	52 (29.6)	66 (37.5)	29 (16.5)	8 (4.6)	1 (0.6)	20 (11.4)
Increased the need to take time off from work to attend caregiving	21 (11.9)	25 (14.2)	45 (25.6)	19 (10.8)	43 (24.4)	23 (13.1)
Put me at a greater disadvantage as a caregiver	34 (19.3)	31 (17.6)	37 (21)	13 (7.4)	38 (21.6)	23 (13.1)
Negatively impacted my focus at work as a caregiver	24 (13.6)	48 (27.3)	34 (19.3)	15 (8.5)	32 (18.2)	23 (13.1)
Prevented me from asking help from others	67 (38.1)	26 (14.8)	28 (15.9)	6 (3.4)	23 (13.1)	26 (14.8)

### Perception of the App

Overall, most respondents had neutral opinions about the apps (Table 4). However, they were more likely to have favorable opinions about decreasing their caregiver burden. For example, 38.1% (67/176) reported that the app made asking others for help with caregiving easier, and 30.1% (53/176) reported that the app enabled them to ask for help for things that they would not have otherwise asked. Of the 176 participants, 74 (42%) reported that the app made them feel more supported in their role as caregivers.

**Table 4:** Responses to questions about perceived value of the app (N=176).

How the app helped	Strongly agree, n (%)	Somewhat agree, n (%)	Neither agree or disagree, n (%)	Somewhat disagree, n (%)	Strongly disagree, n (%)	Missing, n (%)
Asking help for the things I would not otherwise ask	22 (12.5)	31 (17.6)	46 (26.1.7)	22 (12.5)	29 (16.5)	26 (14.8)
Made asking for help easier	27 (15.3)	40 (22.7)	44 (25)	16 (9.1)	23 (13.1)	26 (14.8)
Supported in my role as a caregiver	37 (21)	37 (21)	40 (22.7)	15 (8.5)	21 (11.9)	26 (14.8)
Made caregiving less stressful	26 (14.8)	30 (17)	50 (28.4)	20 (11.4)	24 (13.6)	26 (14.8)

In the second survey, we also asked respondents how caregiving negatively affected their own health. We found that approximately 17% (30/176) said that it affected their physical health and decreased their quality of life. Approximately 26.1% (46/176) and 23.9% (42/176) reported that caregiving worsened their health by affecting their sleep and work-life balance, and 44.9% (79/176) reported that caregiving increased their stress and anxiety (Table 5).

**Table 5:** Responses to questions about the caregiving on caregiver’s health.

How has caregiving affected your own health	Those who answered “Yes,” n (%)
Increased stress or anxiety	79 (44.9)
Affected physical health negatively	29 (16.5)
Affected work-life balance negatively	42 (23.9)
Affected sleep schedule negatively	46 (26.1)
Decreased quality of life	29 (16.5)

## Discussion

### Principal Findings

Our results suggest that mobile app use was associated with improvements in the objective outcomes of caregiving, such as decreasing the likelihood of doing more than half of the caregiving by themselves and the likelihood of the caregiver having no one to assist them. We note that the app works mainly by improving a caregiver’s support system by more conveniently connecting the caregiver to people who can assist with caregiving and making the task of asking for help easier. Therefore, we expected to see relatively more changes in outcomes related to the caregiver’s support system than the other outcomes in this study. We were able to detect significant improvements in outcomes that required objective assessment from the participant, such as the portion of caregiving done on their own versus with assistance. However, outcomes that had subjective measures, such as feeling supported by one’s support group or perceived work productivity, did not yield any meaningful changes.

We recognize that the COVID-19 pandemic brought substantial changes to people’s daily lives and working conditions during the time between the 2 surveys. It is possible that some of the positive and negative outcomes resulted from COVID-19 stay-at-home orders. For example, the increase in the number of people supporting the caregiver could be attributable to more family members working from home or fewer work responsibilities because of a possible job loss, and hence**,** the ability to better support the primary caregiver. Our sample excluded anyone who lost their jobs between surveys; therefore, the results were not affected by a change in the participants’ employment status. The decrease in the number of hours taken off from work to attend to caregiving could be attributed to changes in the participants’ working conditions, not necessarily to the app use. It is also plausible that because of shelter-in-place restrictions, caregivers may not have been able to use the app at its maximum capacity.

The app required users to recruit help from their social networks. Therefore, caregivers without an existing group of potential helpers would not experience the full benefit of the app. The decline in work-related outcomes could have been associated with the effects of the COVID-19 pandemic because the first survey was conducted before the effects of the COVID-19 pandemic were publicly observed, and the second survey occurred during a peak of the COVID-19 pandemic. It is plausible to attribute the decline in perceived work productivity and the increase in feeling disadvantaged at work to the negative impact of the COVID-19 pandemic.

### Limitations

We note that our study sample differs from the samples in many of the studies reviewed in the study by Ploeg et al [15] as well as from most of the other interventions examined in the caregiving domain. First, our study did not restrict the caregiving sample to a particular group of caregivers who provide care to people with specific conditions (ie, heart transplant, patients with cancer, and persons living with dementia) and instead included many types of care recipients. Therefore, we cannot reveal anything to the specific condition of either the care recipient or the caregiver because our sample size was not sufficient for investigating the heterogeneous effects of the app. Second, our sample largely consisted of employees of a large employer, most of whom were at least college educated and had a higher income than the average person. Therefore, our results may not be applicable to the general population of caregivers. It is conceivable that our sample included caregivers with more digital literacy and willingness to use the mobile app compared with the general population, allowing them to reap more benefits. Therefore, our results should be taken with findings from existing literature documenting the link between digital literacy and caregivers’ use of mobile interventions to facilitate caregiving [25,26]. Another limitation of this study is that it only focused on the effect of the app on negative outcomes of caregiving because subjects were not asked anything about their positive experiences attributed to their caregiver role. It is plausible that app use may have enhanced the positive impacts of caregiving by reducing isolation and burden associated with caregiving, leading to more positive experiences attributed to caregivers taking care of their loved ones [11]. Finally, as the intervention period coincided with the emergence of the COVID-19 pandemic, resulting in shelter-in-place policies and other changes brought to people’s daily lives, we must be cautious about interpreting the pandemic’s interference on our findings.

### Conclusions and Implications

We found that using a mobile app that facilitated coordination among caregivers resulted in improvements in the time spent on caregiving, perceived health, and perceived well-being of the primary caregiver. This suggests that mobile interventions to facilitate caregiving can be a useful solution for some caregivers. We believe this study adds further support for devoting resources to mobile apps designed to help caregivers. The implications of our findings are especially relevant as the current pandemic not only increased caregiver burden but also allowed a larger proportion of the population to experience the benefits of technology. Future studies are needed to examine the opportunities that mobile technologies can provide to caregivers with no or limited social support groups and how such technologies can be enhanced to obtain the most help for caregivers in the midst of exceeding demand for unpaid caregiving support.

## Appendix

Multimedia Appendix 1 Caregiving surveys.
