# Supplementary material for: Association Between Mobile App Use and Caregivers’ Support System, Time Spent on Caregiving, and Perceived Well-being: Survey Study From a Large Employer
Source: J Med Internet Res. 2022 Apr 11;24(4):e28504. doi: 10.2196/28504 (PMC9039821; doi:10.2196/28504)
Supplement: Multimedia Appendix 1 [file jmir_v24i4e28504_app1.docx]

**APPENDIX**

Pre Survey

This survey is part of a pilot program conducted. Our goal is to understand the impact caregiving has on employee lives and the opportunity to use technology to support family caregivers, by connecting them to a network of resources for support.

 This survey will take up to 10 minutes. As a reminder, you will receive a **$25 Visa Gift Card** to compensate you for your time only after you complete all requirements in this pilot.
   *Please note: The survey is voluntary and you may end your participation at any time. Please be assured all your responses will remain anonymous. and will be used only for pilot purposes. Survey responses will be combined together without names so no individual is directly identified. All results will be summarized in aggregate. Withdrawing from the survey will not result in any penalty or loss of benefits to which you are otherwise entitled.*

Screening Questions

Do you agree to answer a few questions to see if you qualify?

- Yes
- No

Are you currently employed, either part time or full time?

- Yes
- No

Excluding professionally paid caregiver providers, are you currently the main primary caregiver for at least one person? *(i.e. you manage and coordinate most if not all aspects of a friend or family member’s care. You do not need to be caring for them in your own home.)*

- Yes
- No

Are you willing and able to download an app free of charge on your smart phone or a tablet (Android or Apple IOS)? If you don’t have a suitable smart phone or a tablet please answer “No”.

- Yes
- No

| 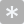 |
| --- |

Please input your first name and email address.

- First name ________________________________________________
- Email address ________________________________________________

*If you are providing caregiver tasks to more than one person, your answers to the next questions should only pertain to the person for whom you spent the most time completing caregiving tasks during the past year.*

Which of the following best describes the person you are caring for?

- Spouse
- Parent
- Child (under the age of 18)
- Adult child (over the age of 18)
- Grandchild
- Grandparent
- Other family member
- Partner
- Friend
- Housemate
- Co-worker
- Neighbor
- Other non-family member

Where does the person you care for live?

- Lives with me
- Lives with their spouse/partner
- Lives with other family
- Lives alone
- Resides in an assisted living facility
- Resides in an independent living facility
- Resides in a nursing home
- Other, please specify ________________________________________________

In the past 30 days, which of the following caregiving tasks did you provide (select all that apply):

- Daily Living Assistance (e.g. food prep, housekeeping, transportation, giving medication, laundry, pet care, shopping, etc.)
- Care Assistance (e.g. feeding, dressing, bathing, grooming, walking, assistance toileting, etc.)
- Admin (e.g. managing insurance, coordinating physician visits, financial management, legal work/decisions, etc.)
- Caregiver self-care (e.g. managing family dynamics, respite care, work balance, etc.)
- Other (please specify) ________________________________________________

In the past 30 days, approximately how many hours per week did you spend on caregiving?

- 0-5 hours
- 6-10 hours
- 11-20 hours
- 21-30 hours
- More than 30 hours

In the past 30 days, how often have you felt overwhelmed by your caregiving tasks (e.g. I worry about the person I care for, I have concerns about how I will manage)

- Never
- Almost Never
- Sometimes
- Fairly Often
- Very Often

In the past 30 days, what portion of your caregiving tasks for this person were done by you even though there may have been other caregivers for this person?  Please select the answer that best reflects your time.

- All of the caregiving for this person was done by me
- More than half of the caregiving for this person was done by me
- Half of the caregiving for this person was done by me
- Less than half of the caregiving for this person was done by me
- Less than 10% of the caregiving for this person was done by me

In the past 30 days, how often have you felt like your role as a Caregiver negatively impacted your own health (e.g. I have missed going to the doctor or taking my own medicine because I am busy caring for someone else)

- Never
- Almost Never
- Sometimes
- Fairly Often
- Very Often

In the past 30 days, aside from any paid caregiver, how many people have supported you in your caregiver tasks? (e.g. food preparation or transportation to a doctor’s visit.)

- No one else
- 1-2 people
- 3-5 people
- More than 5 people

In the past 30 days, how often have you felt supported by your social network (e.g. I feel supported by my friends and family and they make me feel like I’m not alone in my role as a caregiver).

- Never
- Almost Never
- Sometimes
- Fairly Often
- Very Often

In an average month of caregiving, how many hours have you needed to take off work due to previously planned caregiver tasks, such as scheduled doctor appointments?

- 0 hours
- 1-4 hours
- 5-10 hours
- More than 10 hours

In an average month of caregiving, how many hours have you needed to take off work unexpectedly, due to unscheduled caregiver tasks? (e.g. Medical emergencies, no alternative caregiver)

- 0 hours
- 1-4 hours
- 5-10 hours
- More than 10 hours

How do you feel your caregiver role impacts your productivity or focus at work?

- Negatively
- Somewhat Negatively
- Not at all
- Somewhat Positively
- Positively

In the last two years of caregiving, have you requested support from your employer to accommodate your caregiver role? (e.g. alternative work schedule, leave of absence, work from home, information on resources, etc.)

- Yes
- No
- I'm not sure

Do you feel, your employer, is supportive/understanding with regard to your caregiver responsibilities?

- Not supportive
- Somewhat supportive
- Supportive
- Very supportive
- My employer doesn’t know about my caregiving

Do you feel caregiving puts you at a disadvantage compared to your co-workers in terms of work performance and recognition?

- Yes
- Maybe
- No
- I do not know

Please share any additional comments you have about your experience as a family caregiver. Please refrain from sharing information related to your personal health or family history.

________________________________________________________________

________________________________________________________________

________________________________________________________________

________________________________________________________________

________________________________________________________________

These last questions are about you and your background.

Are you…

- Male
- Female
- Other
- Prefer not to respond

| 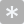 |
| --- |

How old are you?

- Years ________________________________________________

Which one of the following categories best describes your race/ethnicity? *Please indicate only one response.*

- White, non-Hispanic
- Black or African-American, non-Hispanic
- Hispanic or Latino, any race
- Asian, non-Hispanic
- American Indian or Alaska Native, non-Hispanic
- Native Hawaiian or Other Pacific Islander, non-Hispanic
- Other
- Prefer not to respond

What is the highest level of education you have completed or the highest degree you have received? Select one response.

- Less than high school
- Some high school
- High school degree or equivalent (e.g. GED)
- Some college, but no degree
- Associate’s degree, for example 2-year college degree
- Bachelor’s degree, for example B.A. or B.S.
- Graduate degree or higher, for example Master’s
- Prefer not to respond

What is your current marital status?

- Single
- Married
- Domestic Partner
- Separated
- Divorced
- Widowed
- Other
- Prefer not to respond

How many people live in your household including yourself? (Select one answer)

- 1
- 2
- 3
- 4
- 5
- More than 5
- Prefer not to respond

Which of the following categories best describes your household’s total income this past year (2019) before taxes?

- Less than $35,000
- $35,000 - 49,999
- $50,000 - $74,999
- $50,000 - $74,999
- $75,000 - $99,999
- $100K - $124,999
- $125K - $149,999
- More than $150,000 (8)
- Prefer not to respond (9)

Post Survey

This is the second and final part of the survey you took in April, at the beginning of the pilot. Now that you’ve had a chance to use the app, we would like to ask you several questions about your experience with the app and how your caregiver experience has changed in the last several weeks. Finally, we will ask you a few questions about potential new features of the app—your responses could help us upgrade the app to be more helpful to other caregivers. This survey will take approximately 20 minutes.

Please choose the statement that applies to you most accurately.

- I have downloaded the app on my device
- I have not downloaded the app

On a scale from 0-10, how likely are you to recommend the app to a friend or colleague who is also a caregiver?

- 0
- 1
- 2
- 3
- 4
- 5
- 6
- 7
- 8
- 9
- 10

Please enter your “Activity Code” found in the app.
 Open the app, click “settings” on the bottom menu. Select “System Help and Support”. The “Activity Code” is on the bottom right.

- Activity Code ________________________________________________
- I’m having trouble accessing the activity code
- I have not downloaded the app

Your activity code indicates that you onboarded less than 3 people to your care team. Tell us about the reasons your care team was relatively small. Select all that apply:

- COVID-19 made me nervous to ask for outside help
- I did not find the app useful
- The app was giving me technical difficulties
- The people I added weren’t active enough
- I was worried the app would be too complicated for others to use
- I could not find 3 or more people to add from my social group (7)
- Other, please specify: (6) ________________________________________________

| Page Break |  |
| --- | --- |

I have not downloaded the ianacare app because: (select all that apply)

- I forgot to download it
- I was nervous about sharing my data
- It was giving me technical difficulties
- I didn’t think I could add enough people to my “social circle” for it be useful
- COVID-19 made me nervous to ask for outside help
- Other, please specify: ________________________________________________

In a typical week of using the app, how many hours has the app saved you by having someone else from your network sharing the caregiving tasks you would be otherwise responsible?

- The app saved me more than 20 hours per week
- The app saved me 11 to 20 hours per week
- The app saved me 4 to 10 hours per week
- The app saved me 1 to 3 hours per week
- The app did not save me any hours
- The app saved me some time but I am having hard time estimating how much

Please indicate how much you agree or disagree with the following statements:

|  | Strongly agree | Somewhat agree | Neither agree nor disagree | Somewhat disagree | Strongly disagree |
| --- | --- | --- | --- | --- | --- |
| With the app, I asked for help from my support group for things that I would not otherwise ask for help. |  |  |  |  |  |
| The app made asking for help easier / feel like less of a burden |  |  |  |  |  |
| The app made coordinating caregiving tasks easier among my support group |  |  |  |  |  |
| The app made me feel more supported in my role as a caregiver |  |  |  |  |  |
| Using the app made me feel less stressed in my role as a caregiver |  |  |  |  |  |
| COVID-19 prevented me from asking others to help |  |  |  |  |  |

Once the COVID-19 health crisis settles down and the social distancing requirement ends, how will you use the app?

- I would use it more
- I would use it in the same way as during COVID-19
- I would use it less
- I don’t expect to be caregiver anymore

Do you have any comments about your experience with the app you would like to share?

________________________________________________________________

________________________________________________________________

________________________________________________________________

________________________________________________________________

________________________________________________________________

Section II: Now you will be asked about your caregiver experience and how it might have changed over the last several weeks

In the past 30 days, which of the following caregiving tasks did you provide (select all that apply):

- Daily Living Assistance (e.g. food prep, housekeeping, transportation, giving medication, laundry, pet care, shopping, etc.)
- Care Assistance (e.g. feeding, dressing, bathing, grooming, walking, assistance toileting, etc.)
- Admin (e.g. managing insurance, coordinating physician visits, financial management, legal work/decisions, etc.)
- Caregiver self-care (e.g. managing family dynamics, respite care, work balance, etc.)
- Other (please specify) ________________________________________________

In the past 30 days, approximately how many hours per week did you spend on caregiving tasks? (e.g. food prep, care assistance, coordinating physician visits, grocery shopping, etc.)

- 0-5 hours
- 6-10 hours
- 11-20 hours
- 21-30 hours
- More than 30 hours

In the past 30 days, how did the current health crisis due to COVID 19 impact the amount of hours per week you spent on caregiving tasks?

- It significantly increased my caregiving hours
- It somewhat increased my caregiving hours
- It did not have an impact on my caregiving hours
- It somewhat decreased my caregiving hours
- It significantly decreased my caregiving hours

In the past 30 days, how often have you felt overwhelmed by your caregiving tasks (e.g. I worry about the person I care for, I have concerns about how I will manage)

- Never
- Almost Never
- Sometimes
- Fairly Often
- Very Often

In the past month, how did the current health crisis due to COVID 19 impact on your stress levels around caregiving tasks (e.g. I worry about the person I care for, I have concerns about how I will manage)

- It significantly increased my feelings of being overwhelmed
- It somewhat increased my feelings of being overwhelmed
- It did not have an impact on my feelings around caregiving
- It somewhat decreased my feelings of being overwhelmed
- It significantly decreased my feelings of being overwhelmed

In the past 30 days, what portion of your caregiving tasks for this person were done by you even though there may have been other caregivers for this person?  Please select the answer that best reflects your time.

- All of the caregiving for this person was done by me
- More than half of the caregiving for this person was done by me
- Half of the caregiving for this person was done by me
- Less than half of the caregiving for this person was done by me
- Less than 10% of the caregiving for this person was done by me

In the past 30 days, how often have you felt like your role as a Caregiver negatively impacted your own health (e.g. I have missed going to the doctor or taking my own medicine because I am busy caring for someone else)

- Never
- Almost Never
- Sometimes
- Fairly Often
- Very Often

Specifically how has caregiving affected your own health negatively? Choose all that apply.

- By increasing my stress and/or anxiety
- By affecting my physical health
- By negatively impacting my work-life balance
- By negatively impacting my sleep schedule
- By decreasing my life quality
- Other, please specify: ________________________________________________

In the past 30 days, aside from any paid caregiver, how many people have supported you in your caregiver tasks? (e.g. food preparation or transportation to a doctor’s visit.)

- No one else
- 1-2 people
- 3-5 people
- More than 5 people

In the past 30 days, how often have you felt supported by your social network (e.g. I feel supported by my friends and family and they make me feel like I’m not alone in my role as a caregiver).

- Never
- Almost Never
- Sometimes
- Fairly Often
- Very Often

In the past 30 days of caregiving, how many hours have you needed to take off work due to previously planned caregiver tasks, such as scheduled doctor appointments?

- 0 hours
- 1-4 hours
- 5-10 hours
- More than 10 hours

In the past 30 days of caregiving, how many hours have you needed to take off work unexpectedly, due to unscheduled caregiver tasks? (e.g. Medical emergencies, no alternative caregiver)

- 0 hours
- 1-4 hours
- 5-10 hours
- More than 10 hours

How do you feel your caregiver role impacts your productivity or focus at work?

- Negatively
- Somewhat Negatively
- Not at all
- Somewhat Positively
- Positively

Based on your experience in past 30 days, how much do you agree with the following statements:

|  | Strongly agree | Somewhat agree | Neither agree nor disagree | Somewhat disagree | Strongly disagree |
| --- | --- | --- | --- | --- | --- |
| Current health crisis due to COVID 19 has put me at greater disadvantage at work due to being caregiver than I experienced before |  |  |  |  |  |
| ​​​​Current health crisis due to COVID 19 negatively impacted my focus at work because I worry more about caregiving tasks |  |  |  |  |  |
| ​​​​​​Current health crisis due to COVID 19 increased the amount of time I need to take off from work to attend to caregiving tasks |  |  |  |  |  |

Do you feel, your employer, is supportive/understanding with regard to your caregiver responsibilities?

- Not supportive
- Somewhat supportive
- Supportive
- Very supportive
- My employer doesn’t know about my caregiving

Do you feel caregiving puts you at a disadvantage compared to your co-workers in terms of work performance and recognition?

- Yes
- Maybe
- No
- Other, please specify: ________________________________________________

In your last year of caregiving, have you requested support from your employer to accommodate your caregiver role? (e.g. alternative work schedule, leave of absence, work from home, information on resources, etc.)

- Yes
- No
- I'm not sure

Why haven’t you requested support from your employer to accommodate your caregiver role? Select all that apply.

- I did not know that resources were available
- I did not want it to affect my career or productivity perceived by others
- I don’t like to talk about my problems with others
- I did not want anyone to know about my caregiving role
- Other. Please explain:
